# Primary rectal ectopic pregnancy: a rare case report and literature review

**DOI:** 10.3389/fmed.2025.1659535

**Published:** 2025-11-25

**Authors:** Qun Zhang, WenJie Yang, BoWei Wang

**Affiliations:** Department of Obstetrics and Gynecology, The Second Norman Bethune Hospital of Jilin University, Changchun, China

**Keywords:** rectal ectopic pregnancy, extrauterine pregnancy, rare implantation, diagnosis, treatment

## Abstract

**Background:**

Rectal ectopic pregnancy is classified into primary and secondary types, with primary rectal ectopic pregnancy being one of the rarest forms of extrauterine gestation. Currently, there are no standardized guidelines for its diagnosis and treatment.

**Case presentation:**

We present a case of primary rectal ectopic pregnancy in a 37-year-old woman with acute abdominal pain and massive intra-abdominal hemorrhage. Under the premise of gynecological ultrasound suggesting conventional tubal or ovarian ectopic pregnancy, we employed diagnostic single-port laparoscopy to exclude typical adnexal pregnancies. Through comprehensive exploration of both the upper and lower abdominal cavities, we ultimately confirmed the diagnosis of primary rectal ectopic pregnancy. And through coordinated efforts with gastrointestinal surgeons, we performed precise resection of the ectopic gestational tissue while preserving rectal integrity, with prompt control of intra-abdominal hemorrhage. This emergency intervention achieved dual success: lifesaving management coupled with minimally invasive advantages, Enhanced recovery after surgery and resulting in a nearly invisible umbilical incision with optimal cosmetic outcomes.

**Conclusion:**

Patients with rectal ectopic pregnancy often present with nonspecific early symptoms, leading to frequent misdiagnosis or delayed diagnosis. Definitive diagnosis is typically established only after the onset of severe complications. Through a multidisciplinary approach to this rare case and a systematic review of the literature, we have derived the following important clinical insights: 1. Early diagnosis strategy: early identification of REP requires a multimodal approach, including dynamic monitoring of serum β-hCG levels, pelvic ultrasound, and multimodal imaging assessment with CT/MRI. Diagnostic laparoscopy should be performed when necessary to confirm the location and extent of the lesion. 2. Broadening differential diagnosis considerations: in all women of childbearing age who are considered to be pregnant, especially when intrauterine, tubal, or ovarian pregnancy is not detected by ultrasound, REP should be included in the differential diagnosis. 3. Value of Minimally Invasive Surgery: Single-port laparoscopic surgery has demonstrated comprehensive advantages in such emergency surgeries, including minimal invasiveness, rapid recovery, and cosmetic benefits. However, this technique requires the surgeon to possess advanced skills, and the patient must meet the indications for single-port laparoscopic surgery. This article also provides important reference evidence for the development of standardized diagnostic and treatment protocols for rectal ectopic pregnancy.

## 1 Background

Ectopic pregnancy remains a significant contributor to maternal morbidity and mortality worldwide, representing 1.3% to 2% of all pregnancies (1, 2). This condition is characterized by the implantation of a fertilized ovum outside the uterine cavity, with the fallopian tubes being the most common site, accounting for approximately 95% of cases (3). Among the various forms of ectopic pregnancy, rectal ectopic pregnancy (REP) is exceedingly rare. Patients with REP often exhibit nonspecific early clinical symptoms, which frequently lead to misdiagnosis as a biochemical pregnancy or result in diagnostic oversight, as pregnancy-related tissue are not detected within the uterus or bilateral adnexa (4). In the absence of ongoing serum beta-human chorionic gonadotropin (β-hCG) monitoring, REP is typically diagnosed only when severe complications emerge, such as intraperitoneal hemorrhage, intestinal obstruction, enterocutaneous fistula, or rectal bleeding (5–7). These complications can markedly elevate the risk of maternal mortality. It is estimated that REP occurs in approximately 10 cases per 100,000 pregnancies (8), with a reported maternal mortality rate ranging from 2% to 30% (9). REP is highly challenging to diagnose and manage. Early diagnosis not only prevents severe complications and life-threatening events but also offers a wider range of therapeutic options. Clinicians should integrate detailed clinical history, imaging modalities (such as ultrasound, CT, MRI), continuous monitoring of serum β-hCG levels, or diagnostic laparoscopy. Particularly when ectopic pregnancy is suspected but no tubal or ovarian pregnancy is detected during diagnostic laparoscopy, gynecologists should be vigilant for the possibility of a fertilized ovum implanting in the rectum (10). In terms of treatment, for early-stage REP without contraindications, conservative management with local or systemic methotrexate therapy can be considered (11). For late-stage, critical, or drug-resistant REP, surgical intervention is warranted, including laparoscopic surgery (multiport or single-port) and laparotomy. In all cases, a multidisciplinary team comprising gynecologists and gastrointestinal surgeons is essential for managing the gestational tissue in the rectum or rectal injuries. Through systematic evaluation of this case and synthesis of existing literature, we present the first reported use of single-port laparoscopic technique for the diagnosis and treatment of rectal ectopic pregnancy, comprehensively outlining the diagnostic approaches and management algorithms for this condition, thereby offering valuable references for clinical practice. Studies have shown (12) that in colorectal resections, single-port laparoscopy offers its principal advantage over conventional multi-port approaches in markedly reduced invasiveness and superior cosmesis. By performing complex procedures through a single incision, the technique not only lessens surgical trauma and post-operative pain but also conceals the scar, greatly improving aesthetic satisfaction. Current evidence indicates (12) that although the two approaches yield broadly comparable major surgical outcomes, the minimally invasive character of single-port laparoscopy secures its important clinical niche. Looking forward, the method dovetails with enhanced-recovery-after-surgery (ERAS) protocols, opening prospects for optimised peri-operative care and accelerated patient recovery. Nevertheless, recommending it as the standard replacement for multi-port laparoscopy awaits more robust evidence from well-designed trials.

## 2 Case presentation

**Figure 1:**
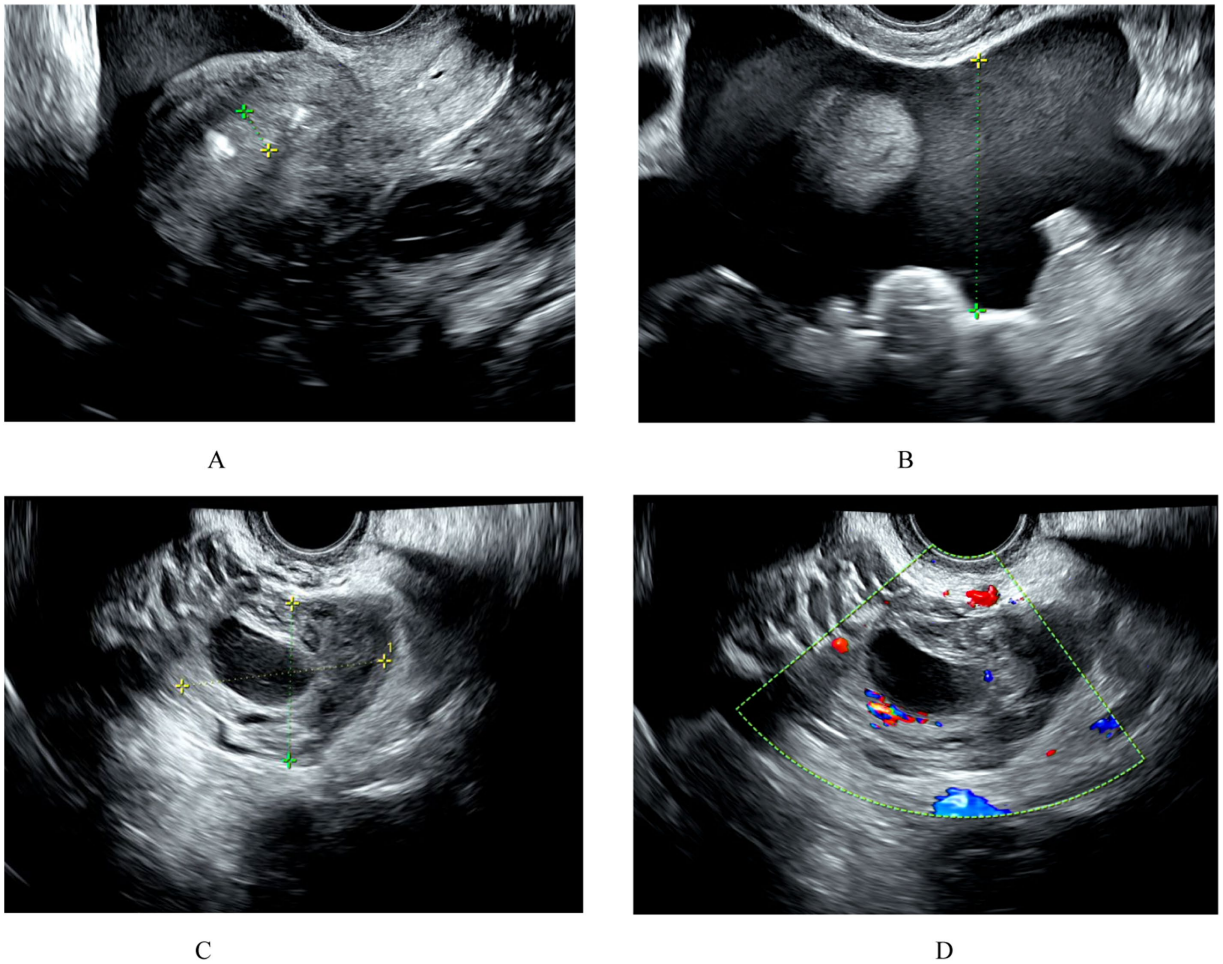
**(A)** The endometrium is thickened to 1.1 cm. An IUD is visible in the uterus, and its position is correct. No gestational sac is seen in the uterus. **(B)** Hemorrhagic fluid is visible in the pelvic cavity. **(C,D)** A mixed echo of 5.1 × 3.6 cm is seen in the left adnexal region, and ectopic pregnancy is suspected on ultrasound.

The patient is a 37-year-old female with a regular menstrual cycle. She was admitted to the emergency department due to amenorrhea for over 30 days, lower abdominal pain for 1 h, and worsening pain for 5 h. Her medical history includes one cesarean section and one induced abortion. Upon admission, her vital signs showed a blood pressure of 90/60 mmHg and a heart rate of 110 beats per minute. The relevant examination results are as follows:

(1) Transvaginal ultrasound examination results: Endometrial thickness: the endometrium is thickened, measuring 1.1 cm. An intrauterine device (IUD) is visible within the uterine cavity, with normal positioning. Two hypoechoic areas are observed in the posterior wall of the uterus, with the larger one measuring 1.5 cm × 1.1 cm. A mixed echogenicity mass measuring 5.1 cm × 3.6 cm is seen in the left adnexal region. An anechoic area measuring 2.2 cm × 1.7 cm is present in the right adnexal region. An inhomogeneously hyperechoic area measuring 7.2 cm × 4.1 cm is observed in the pelvic cavity (considered to be a hematoma). Free fluid is noted in the hepatorenal recess (4.6 cm), splenorenal recess (2.9 cm), and pelvic cavity (5.5 cm) (see Figure 1).
(2) Abdominal ultrasound examination results: Free fluid is detected in the abdominal cavity, with a depth of approximately 3.0 cm around the liver and 3.5 cm in the intestinal space of the lower abdomen.
(3) Serum β-hCG level: 1267.87 mIU/ml.
(4) Coagulation profile: Activated Partial Thromboplastin Time (aPTT): 24.1 s (↓) [Reference range: 25.1–36.5], Partial Thromboplastin Time Ratio: 0.76 (↓) [Reference range: 0.86–1.25], D-Dimer: 1.75 mg/L (↑) [Reference range: 0–0.5], Fibrinogen Degradation Products (FDP): 4.53 mg/L (↑) [Reference range: 0–2.01].

Based on detailed medical history, transvaginal ultrasound, abdominal ultrasound, serum β-hCG levels, and other relevant examinations, an initial diagnosis of ectopic pregnancy was made. Given the patient’s clinical presentation of hypotension (90/60 mmHg) and tachycardia (110 beats per minute), suggestive of acute internal bleeding, an urgent surgery was planned to confirm the diagnosis and provide appropriate treatment.

**Figure 2:**
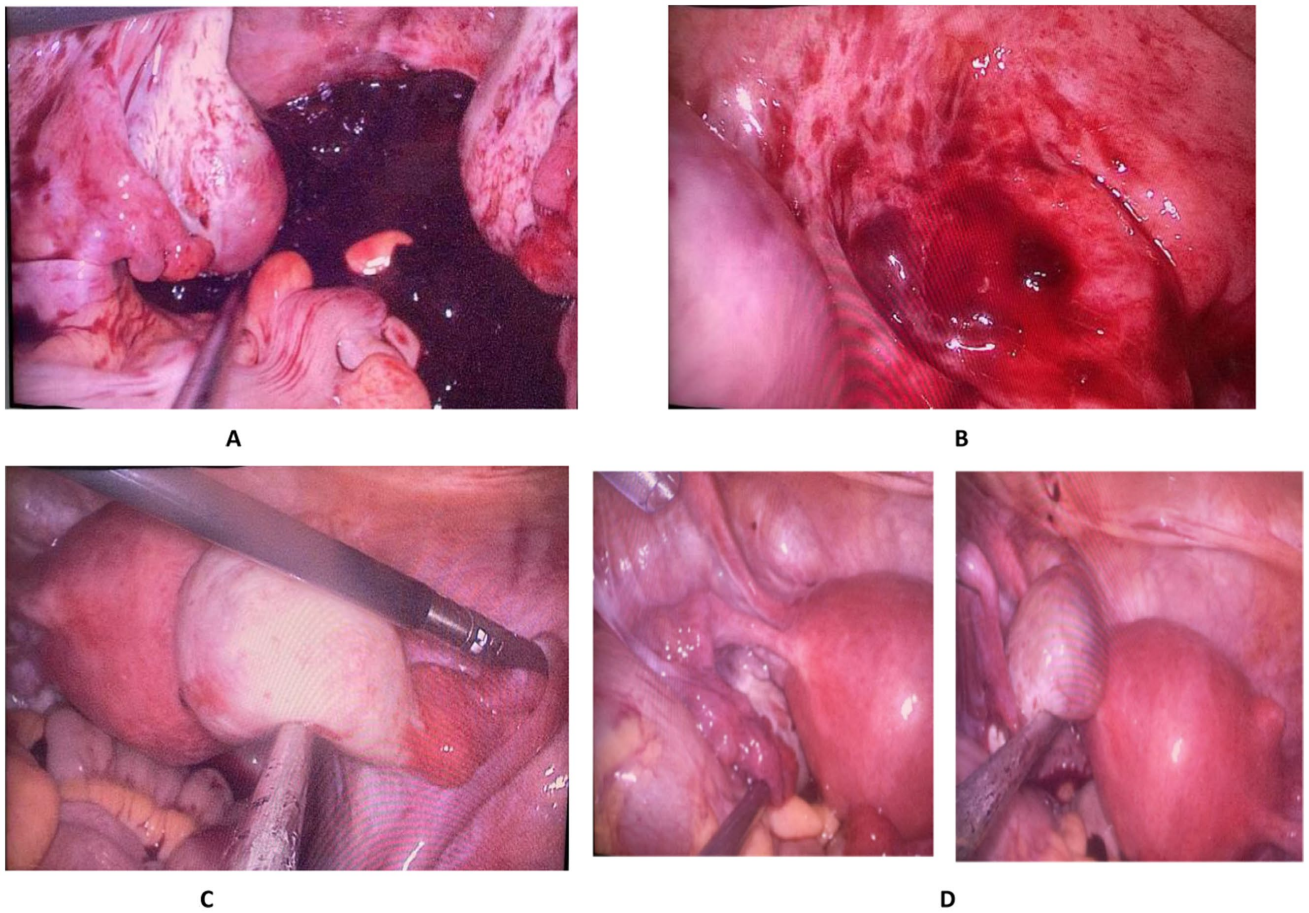
**(A)** Hemoperitoneum in the pelvic and abdominal cavities; **(B)** Bleeding site on the rectum; **(C)** The right fallopian tube and ovary show no bleeding sites or gestational lesions, with a corpus luteum cyst visible on the right ovary. **(D)** The left fallopian tube and ovary show no bleeding sites or gestational lesions, with a corpus luteum cyst visible on the right ovary.

We planned a single-port laparoscopic surgery for the patient. The umbilicus was selected as the surgical incision site, with an incision length of approximately 1–2 cm. A “KANGJI” single-port disposable trocar was used for access. Intraoperatively, approximately 1,000 mL of hemoperitoneum was found in the pelvic and abdominal cavities (Figure 2A). After aspiration of the hemoperitoneum, exploration revealed cysts on both ovaries, measuring approximately 2.5 cm and 3.0 cm in diameter, respectively, which appeared to be corpus luteum cysts. The bilateral fallopian tubes appeared normal without any obvious abnormalities. No bleeding points were identified on either the ovaries or the fallopian tubes (Figures 2C,D). Additionally, a subserosal nodule, approximately 1 cm in size, was observed at the fundus of the uterus.

In this scenario, we initially ruled out the conventional possibilities of tubal or ovarian ectopic pregnancy and considered the rare occurrence of an ectopic pregnancy in the abdominal cavity or bleeding from other intra-abdominal organs. Consequently, we invited a general surgeon to perform a comprehensive exploration of the upper abdomen, including the liver and spleen, without identifying any bleeding sites. Subsequently, a thorough exploration of the lower abdomen revealed a gestational tissue implantation on the surface of the rectum, with active bleeding observed (Figure 2B).

Following the definitive diagnosis of primary rectal ectopic pregnancy, we first introduced povidone-iodine into the rectum via anal irrigation. Under laparoscopic surveillance, it was confirmed that no povidone-iodine leakage into the peritoneal cavity occurred from the rectal lesion. The gestational tissue was identified to be located on the rectal serosal surface, and excision of the gestational mass was performed. During the procedure, the integrity of the rectal muscular layer was deliberately preserved, and no rectal wall resection was carried out. Throughout the surgery, no leakage of povidone-iodine into the abdominal cavity was observed. Additionally, concurrent management of bilateral ovarian cysts and a subserosal uterine myoma was performed. The entire procedure was completed without causing any injury to the rectum.

The patient recovered well postoperatively. Serial serum β-hCG measurements showed a decline to 317.1 mIU/mL on postoperative day (POD) 1, 126.9 mIU/mL on POD 3, and 65.2 mIU/mL on POD 5, reaching the normal range by POD 14.

**Figure 3:**
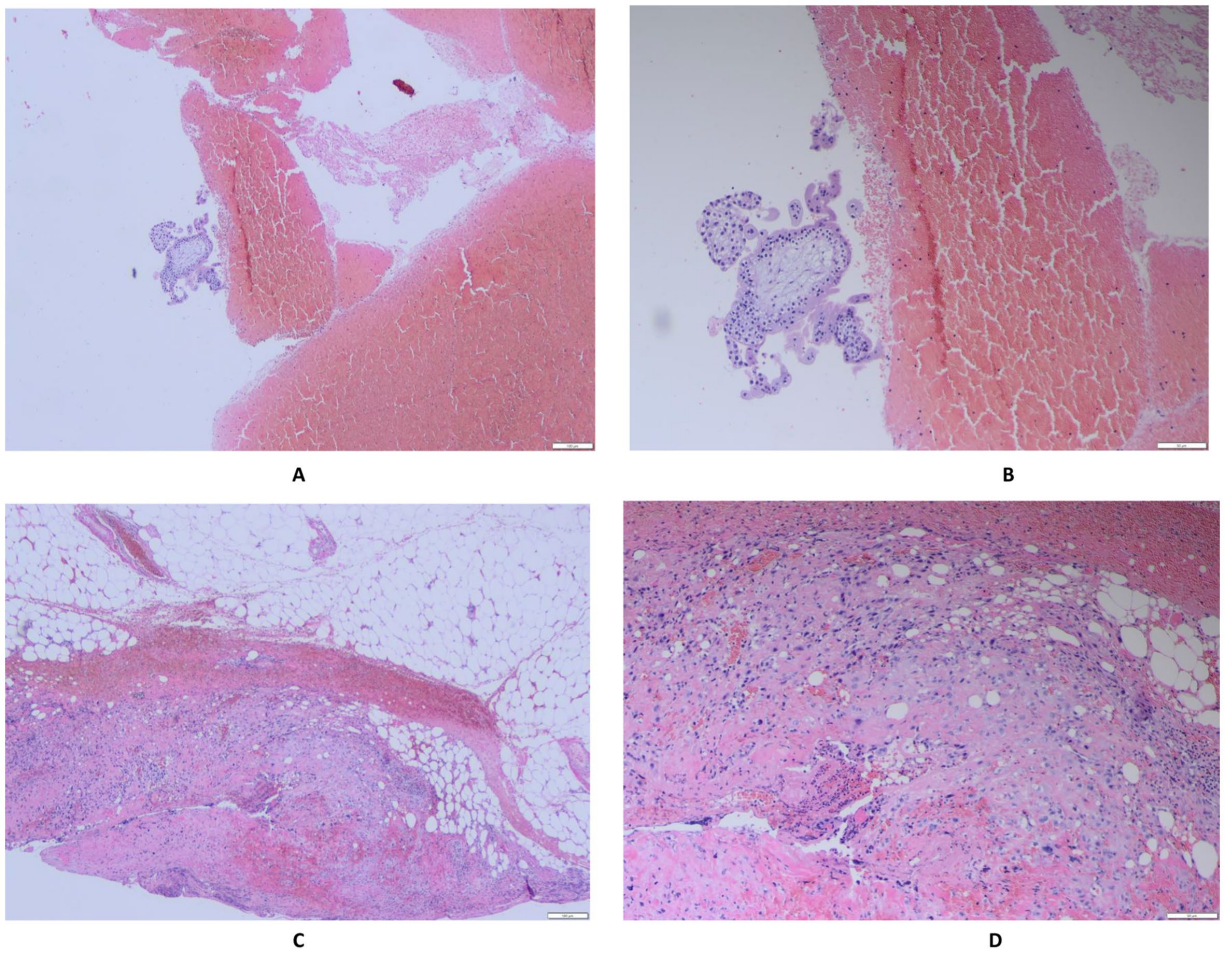
**(A,B)** (Pelvic hematoma) Chorionic villi and infiltrating trophoblastic cells are observed. **(C,D)** (Gestational tissue attached to the rectum) Infiltrating trophoblastic cells are present.

Postoperative pathological examination showed that: (gestational tissue adherent to the rectal serosa) contains trophoblast cells; (pelvic blood clot) contains chorionic villi and trophoblast cells; (uterus) serosal leiomyoma; (ovaries) bilateral luteal cysts. (Figure 3).

## 3 Discussion and conclusion

REP represents a rare subtype of abdominal ectopic gestation, which can be pathologically classified into primary and secondary types. Current evidence suggests (13) that the majority of reported cases are secondary rectal ectopic pregnancies, predominantly resulting from secondary implantation of embryos following tubal rupture or tubo-abdominal abortion. In 1942, Studdiford (14) established the diagnostic criteria for primary abdominal pregnancy, which include: 1. Normal fallopian tubes and ovaries, without evidence of recent or prior injury. 2. No demonstrable utero-peritoneal fistula. 3. A pregnancy confined exclusively to the peritoneal surface, at an early gestational stage that precludes the possibility of secondary implantation following initial tubal nidation. In the present case, the uterus and bilateral fallopian tubes and ovaries were normal, with no evidence of rupture or bleeding sites. The anterior wall of the rectum exhibited a distinct bleeding site with attached gestational tissue, which is consistent with the diagnostic criteria for primary REP. Literature review indicates (4, 10, 11, 15) that ectopic gestational tissue typically adheres only to the rectal serosal surface, with muscularis invasion being uncommon. However, exceptions exist: one case (10) described a large lesion that had infiltrated deeper layers, necessitating intra-operative rectal wall repair. We postulate that this deep invasion resulted from delayed diagnosis, which allowed the gestational tissue prolonged time to grow and infiltrate. Therefore, strictly defining REP as an early-stage lesion confined to the rectal serosa is inaccurate; available evidence (10) indicates that delayed diagnosis allows trophoblastic tissue to penetrate the muscularis and even involve the mucosa. Consequently, the clinical spectrum should encompass everything from serosal adherence to full-thickness infiltration.

The high-risk factors for REP include a history of ectopic pregnancy, history of tubal surgery, history of cesarean section, use of assisted reproductive technology, pelvic inflammatory disease, smoking, and contraceptive failure (such as failure of IUD contraception or emergency oral contraception) (16). The present case is associated with multiple high-risk factors, including a history of one cesarean section and failure of IUD contraception. The presence of an IUD also led the patient to disregard the possibility of pregnancy until she experienced acute abdominal pain with hemoperitoneum, prompting her to seek medical attention. There are also reports of rectal ectopic pregnancy in women undergoing *in vitro* fertilization (10, 11).

**Figure 4:**
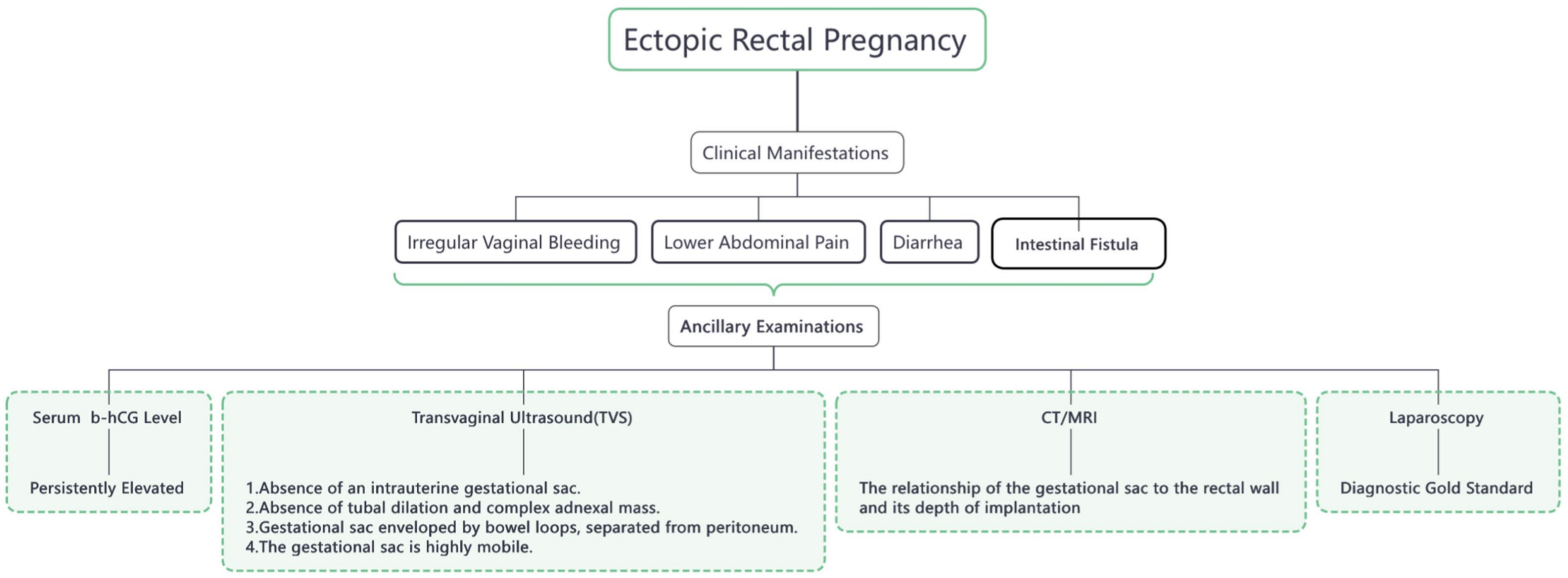
Strategies for early identification of rectal ectopic pregnancy.

As shown in Figure 4, the clinical manifestations of REP are nonspecific and can present with a variety of symptoms, including abdominal pain, diarrhea, vaginal bleeding, menstrual irregularities, and even complications such as enteric fistula or intestinal obstruction (5, 11, 15, 17–19). These factors also increase the difficulty of diagnosing REP. Early diagnosis is of great significance as it can maximize the reduction of the risk of severe complications and mortality in pregnant women. Currently, the combination of transvaginal ultrasound and serum β-hCG concentration measurement is the preferred method for diagnosing REP. For pregnancies with an unknown location, especially when the gestational sac is not detected in the uterus and bilateral fallopian tubes and ovaries by ultrasound, and the serum β-hCG concentration continues to rise, CT, MRI, or diagnostic laparoscopy can be considered to aid in diagnosis. Although ultrasound is the first-line method for diagnosing rectal pregnancy, its sensitivity varies widely, ranging from 50% to 90% (20). The current ultrasound criteria for diagnosing REP include (21, 22): 1. Absence of an intrauterine gestational sac. 2. Absence of both a clearly dilated fallopian tube and a complex adnexal mass. 3. A gestational cavity surrounded by loops of bowel and separated by peritoneum. 4. A wide mobility similar to fluctuation of the sac, particularly evident with gentle pressure of the transvaginal probe toward the posterior cul-de-sac. CT and MRI are generally capable of delineating the relationship between the gestational sac and the rectal wall, as well as the depth of implantation (10), thereby facilitating the formulation of precise diagnostic and treatment plans. Diagnostic laparoscopy can be regarded as the “gold standard” for diagnosing REP. However, a comprehensive and meticulous exploration is of paramount importance to avoid misdiagnosis and missed diagnosis (4, 10), thereby ensuring patient health and safety. The patient reported here presented without any early symptoms and sought medical attention only after experiencing sudden abdominal pain that progressively worsened. At the time of presentation, the serum β-hCG level was 1267.87 mIU/ml, and ultrasound examination revealed a mixed echogenic mass in the left adnexal region. The patient had already developed hypotension and tachycardia. Although hemoglobin levels had not yet significantly decreased due to compensatory mechanisms, coagulation dysfunction was already present. Without timely intervention, the patient was at risk of developing hemorrhagic shock, which could have been life-threatening. Based on the initial consideration of a conventional tubal or ovarian ectopic pregnancy, we planned a single-port laparoscopic diagnostic procedure. However, no gestational sac was identified in the bilateral fallopian tubes or ovaries during surgery. At this point, a comprehensive exploration was of utmost importance. Ultimately, in collaboration with general surgery, we confirmed the diagnosis of primary REP.

There is a paucity of data regarding the optimal treatment strategy for REP, and no standardized treatment protocol currently exists. However, surgical intervention remains the primary therapeutic approach (23). For early-stage REP, when there are no contraindications to medical therapy, methotrexate can serve as a conservative treatment alternative to surgery (11). Surgical options encompass laparoscopic surgery (both single-port and multi-port) and laparotomy. In cases of severe or critical REP, laparotomy may be the preferred choice, as it does not require advanced laparoscopic skills and allows for more effective management of the REP and control of bleeding (21, 24, 25). Certainly, if the surgical team possesses extensive laparoscopic experience—encompassing both single-port and multi-port techniques—laparoscopic surgery can be the first-line approach for both diagnosis and treatment. In this case, we employed single-port laparoscopy, which enabled faster postoperative recovery and an almost scarless incision. This approach embodies the modern surgical trend of “less trauma, better cosmesis, and higher satisfaction,” aligning perfectly with the patient-centered principles of enhanced recovery.

A pooled analysis of the cases in Table 1 shows that REP—a rare form of ectopic implantation—presents with non-specific symptoms such as abdominal pain, diarrhoea, vaginal bleeding or tenesmus that are easily mistaken for enteritis, appendicitis, pelvic inflammatory disease or ovarian torsion. IUD or lactation-related menstrual irregularities often further delay presentation. Although serial serum β-hCG measurements are helpful, a single value neither excludes nor confirms REP. Imaging is equally challenging. The classic trans-vaginal ultrasound triad—empty uterus, no adnexal mass and a free-floating gestational sac in the pouch of Douglas—is present in only a minority of cases, and the false-negative rate can reach 10%–50%. Accuracy depends heavily on operator experience and repeated meticulous scanning. CT or MRI can precisely depict the relationship between the gestational sac and the rectal wall, but these examinations are frequently unavailable in the emergency setting, resulting in persistently high misdiagnosis rates and, not infrequently, a second surgical procedure, substantially increasing patient morbidity and healthcare risk. Management is fraught with additional hazards. Management is dictated by the exact anatomical relationship between the gestational tissue and the rectum. Under multidisciplinary-team (MDT) guidance the goal is complete excision of the ectopic conceptus, eliminating any possibility of residual villi, with or without concurrent suture repair of the rectal wall. Postoperative serum β-hCG levels are monitored closely, and adjuvant medical therapy is given if necessary. Systemic or local MTX offers a non-surgical alternative, but is suitable only for haemodynamically stable, unruptured, early REP with serum β-hCG < 5,000 IU/L. Given the paucity of cases, dosing protocols, treatment duration and monitoring strategies are all extrapolated from tubal ectopic pregnancy, and the balance between efficacy and safety remains unsupported by REP-specific data.

**Table 1:** Comparative analysis of clinical characteristics and treatment outcomes in rectal ectopic pregnancies: a literature review.

Feature	Case 1 (10)	Case 2 (15)	Case 3 (11)	Case 4 (4)
Age/obstetric history	32 years old, G2P1	32 years old, G1P0	20 years old, G3P2	25 years old, G2P1
Risk factors	One previous cesarean delivery	IVF-ET	Two previous cesarean deliveries	Lactation period, no contraception
Initial symptoms	Vaginal bleeding + mild abdominal pain	Lower abdominal pain + diarrhea + anal fullness + dysuria	Abdominal pain + amenorrhea	Lower abdominal pain + nausea
Initial β-hCG + Ultrasound	At 6 weeks of gestation, the serum β-hCG level was 7,000 IU/L and ultrasound examination revealed the absence of a gestational sac within the uterine cavity.	On the 11th day following IVF-ET, the serum β-hCG concentration was 2.31 mIU/mL. Ultrasound imaging failed to detect any dominant follicle development.	—	Considered ectopic pregnancy (location undetermined)
Initial misdiagnosis	Initially diagnosed as ectopic pregnancy, but no gestational sac was found during laparoscopy.	Initially diagnosed as biochemical pregnancy	Initially diagnosed as ovarian ectopic pregnancy	Initially diagnosed as ectopic pregnancy, but conservative treatment failed
Key imaging	Persistently elevated serum β-hCG levels were observed postoperatively, rising from 12,000 IU/L at 7 days to 16,088 IU/L at 12 days. Transvaginal ultrasound revealed two adjacent masses adjacent to the rectal wall: A 19 × 17 mm mass containing a yolk sac (indicating embryonic tissue viability) An irregular hypoechoic mass (26 × 17 mm) with heterogeneous echogenicity No intrauterine gestational sac was identified.	At 40 days following in vitro fertilization and embryo transfer (IVF-ET), serum β-hCG measured 12,451.6 mIU/mL. Abdominal ultrasonography revealed: Endometrial thickness of 5 mm with no identifiable intrauterine gestational sac; A moderate amount of free fluid in the pelvic cavity; A sac-like mass (54 × 24 mm) posterior to the uterus.	Five days after laparoscopy, the patient’s β-hCG level continued to rise. Through MRI and pelvic ultrasound, a gestational sac attached to the rectal wall was clearly detected.	The patient abruptly developed fatigue, pallor, and severe abdominal pain. Vital signs revealed hypotension (90/60 mmHg) and tachycardia (120 beats/min). Laboratory findings demonstrated a significant hemoglobin drop to 7.9 g/dL (normal range: 12.3–15.3 g/dL). Ultrasonography showed a normal-sized uterus with regular endometrial thickness, while the abdominal cavity was filled with fluid and clots extending to Morison’s pouch
Treatment method	Repeated laparoscopic surgery	Laparoscopic surgery	Local methotrexate (MTX) was injected under ultrasound guidance, combined with a systemic MTX regimen administered intramuscularly	Open abdominal surgery
Intraoperative findings	A 3 × 3 cm gestational sac was found in the right Douglas. The placental tissue was implanted on the anterior wall of the rectum.	Following aspiration of approximately 1,000 mL of hemoperitoneum and blood clots, a ruptured mass was identified in the pouch of Douglas. Additionally, an actively bleeding mucosal laceration (25 mm) was observed on the anterior rectal wall.	—	A large amount of blood and clots were seen. Residual placental and fetal tissue was found in the upper third of the anterior wall of the rectum.
Multidisciplinary collaboration	A 1 cm defect was found in the rectal wall after placental removal. The gastrointestinal surgeon sutured the rectal mucosa.	None	Multidisciplinary consultation	The surgeon examined the rectum and found no damage or perforation, while also removing the inflamed appendix.
Time to β-hCG negative	2 months after surgery	25 days after surgery	—	3 weeks after surgery
Severe complications	None	Blood loss of 1,000 mL	—	Hemorrhagic shock

REP represents an extremely rare and potentially life-threatening form of ectopic gestation. Due to its nonspecific clinical manifestations and the lack of standardized diagnostic criteria, this condition poses significant challenges in clinical management. Multidisciplinary team (MDT) approach plays a pivotal role in achieving accurate diagnosis and tailored treatment. In this case, our team successfully performed single-port laparoscopic surgery, demonstrating the feasibility and advantages of minimally invasive techniques in managing such rare entities. This approach not only ensured precise surgical intervention but also contributed to rapid postoperative recovery and nearly scarless wound healing. Given the clinical rarity of REP, there is an urgent need to consolidate case reports and clinical data worldwide to facilitate the development of standardized diagnostic and therapeutic guidelines. By systematically synthesizing clinical experience and evidence-based findings, we can enhance physicians’ ability to recognize REP and standardize its management, thereby minimizing misdiagnosis and missed diagnosis, and ultimately improving patient outcomes.

## Data Availability

The raw data supporting the conclusions of this article will be made available by the authors, without undue reservation.
